# FMC^2^ model based perception grading for dark insurgent network analysis

**DOI:** 10.7717/peerj-cs.1644

**Published:** 2023-12-05

**Authors:** Ganesh Kumar Pugalendhi, Shanmugapriya Kumaresan, Anand Paul

**Affiliations:** 1 Department of Computer Science and Engineering, College of Engineering, Guindy, Anna University, Chennai, Tamilnadu, India; 2 Advanced Analytics Department, Indium Software (India) Private Ltd, Chennai, Tamilnadu, India; 3 The School of Computer Science and Engineering, Kyungpook National University, Daegu, South Korea

**Keywords:** Dark network, Social network analysis, Influential nodes, MCMC decision making, Perception-based grading, Sensitivity analysis, Data science

## Abstract

The burgeoning role of social network analysis (SNA) in various fields raises complex challenges, particularly in the analysis of dark and dim networks involved in illicit activities. Existing models like the stochastic block model (SBM), exponential graph model (EGM), and latent space model (LSM) are limited in scope, often only suitable for one-mode networks. This article introduces a novel fuzzy multiple criteria multiple constraint model (FMC^2^) tailored for community detection in two-mode networks, which are particularly common in dark networks. The proposed method quantitatively determines the relationships between nodes based on a probabilistic measure and uses distance metrics to identify communities within the network. Moreover, the model establishes fuzzy boundaries to differentiate between the most and least influential nodes. We validate the efficacy of FMC2 using the Noordin Terrorist dataset and conduct extensive simulations to evaluate performance metrics. The results demonstrate that FMC2 not only effectively identifies communities but also ranks influential nodes within them, contributing to a nuanced understanding of complex networks. The method promises broad applicability and adaptability, particularly in intelligence and security domains where identifying influential actors within covert networks is critical.

## Introduction

In an interconnected world, social networks serve as intricate tapestries where diverse entities—ranging from individuals and organizations to digital domains—engage to realize shared objectives. Social network analysis (SNA) has emerged as an indispensable tool for decoding these complex webs, offering quantitative insights into the relationships and interactions that define these networks (Everton & Roberts, 2011). Within the taxonomy of SNA, networks can be broadly categorized into light, dim, and dark, each with distinct characteristics and implications. Light networks are transparent and open, fostering benign activities. Dim networks, while not overtly secretive, maintain a guarded interface with external organizations. Dark networks (Milward & Raab, 2003; Rawat et al., 2021), however, operate in the underbelly of society, facilitating illicit activities such as drug trafficking, money laundering, and terrorism, and thus pose challenges for comprehensive analysis due to their concealed and dynamic nature. In the realm of intelligence analytics, social network analysis (SNA) serves as a transformative lens, fundamentally altering how analysts decipher intricate networks. While traditional networks are essentially extensions of offline social circles—comprising individuals bound by pre-existing relationships and shared activities—the landscape dramatically shifts when navigating dark networks. These clandestine networks are nebulous entities, characterized by incomplete data, ambiguous relationships, and a volatile structure, all of which defy straightforward analysis. Current methodologies in SNA predominantly rely on statistical models (Kolaczyk, 2009) such as the stochastic block model (SBM) (Holland, Laskey & Leinhardt, 1983), exponential graph model (EGM), and latent space model (LSM). While SBM pioneered community detection within networks, subsequent adaptations, notably in exponential random graph models (Frank & Strauss, 1986; Robins et al., 2007), have refined the understanding of dynamic networks. However, these models have been largely optimized for one-mode networks and falter when applied to more complex structures.

In contemporary network science, a paradigm shift is observed where networks are projected into latent spaces. This projection is primarily grounded on probabilistic assessments of inter-node relationships, further refined by distance metrics (D’Angelo, Alfò & Fop, 2023). Recent advancements in LSM have transcended static networks to accommodate their dynamic evolution through iterative modifications in distance measures (Handcock, Raftery & Tantrum, 2007; Sewell, Chen & Etal, 2017). However, these innovations are predominantly tailored for one-mode networks, where nodes share homogenous characteristics, thus limiting their applicability in more complex scenarios.

To address this gap, the present study introduces a novel fuzzy multiple criteria multiple constraint model (FMC^2^), specifically designed for dissecting two-mode networks. The methodology employs probabilistic estimations of node relationships coupled with distance metrics to delineate communities within the network. Moreover, it establishes perceptual boundaries to segregate best-case and worst-case nodes within these communities, thereby identifying the most influential nodes in a hierarchical fashion. The efficacy of this perception-based grading approach has been rigorously validated using the Noordin Terrorist dataset, a publicly accessible benchmark. A series of simulations further corroborate the model’s precision and robustness in both community identification and influence ranking.

The ensuing sections of this article are meticulously structured to furnish a comprehensive understanding of the research underpinning. ‘Social Network Analysis’ delves into the foundational principles and metrics germane to social network analysis, serving as a primer for the uninitiated. ‘Model Selection’ elucidates existing models, laying the groundwork for an appreciation of the limitations that this research seeks to overcome. ‘Analytical Framework for Bipartite Insurgent Network Dissection’ unveils the architectural blueprint of the proposed methodology, providing an aerial view of the research landscape. ‘Comprehensive Execution of FMC^2^ Model for Analyzing Insurgent Networks’ offers a granular walkthrough of the implementation steps for the novel FMC2 model-based perception grading, explicating the mechanisms for community and influential node identification. ‘Simulation Result’ presents empirical evidence, showcasing the results derived from applying the proposed algorithm to real-world datasets, complemented by performance metrics. Finally, ‘Conclusion and Future Work’ synthesizes the overarching conclusions while charting a roadmap for future explorations in this intriguing domain.

**Figure 1:**
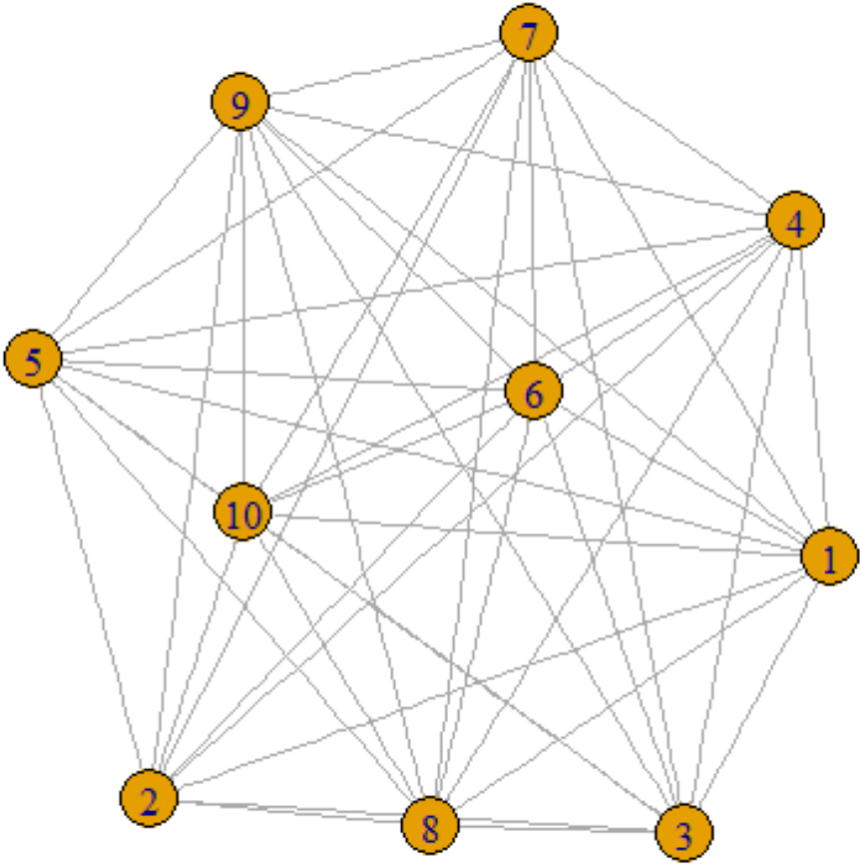
One-mode projection network.

## Social Network Analysis

Social network analysis (SNA) is fundamentally a graph-theoretical method, wherein vertices represent various types of actors—be they individuals, organizations, or other entities—and edges encapsulate the relationships among these vertices. Typically, networks can be categorized into one-mode and two-mode projections. In a one-mode projection, the network is formally represented as *SG* =*{V,E},* where *V* denotes the set of vertices and *E* the set of edges. Such representations are commonly employed for modeling social media interactions, such as friendships on Facebook, as illustrated in Fig. 1. In these networks, nodes are labeled numerically as 1, 2, 3, up to 10, and edges are represented by lines that connect these nodes.

**Figure 2:**
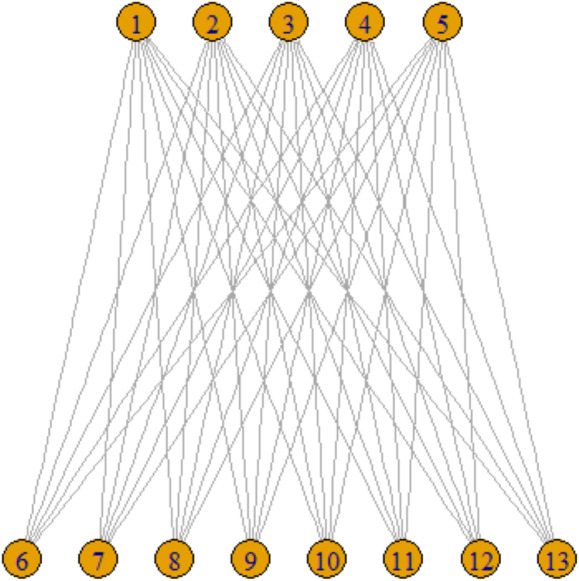
Two-mode projection network.

However, one-mode projections are ill-suited for capturing the nuances of specific types of networks, such as scientific collaboration networks, where relational ties are more complex. For instance, an edge may exist between two authors if they have co-authored a paper. This leads to the concept of a two-mode, or bipartite, network. In such networks, formally represented as *BG* =*{U,V,E* }, vertices are divided into two distinct sets *U* and *V,* with edges *E* connecting vertices across these sets but not within them, as depicted in Fig. 2. These bipartite structures are particularly apt for modeling affiliation and bibliographic networks. This type of network is also called as affiliation network.

A multitude of metrics serve as the analytical linchpins in deciphering the complex tapestry of network structures. Among these, centrality measures—encompassing degree, closeness, betweenness, and hub scores—are particularly salient (Hansen et al., 2019). The subsequent sections delve into the specific centrality metrics employed in this scholarly inquiry, beginning with degree centrality. Degree centrality: regarded as a cornerstone metric, degree centrality is fundamentally a quantitative measure that enumerates the ties a node maintains within the network. This attribute serves as an indicator of a node’s relational intensity and is mathematically formalized in Eq. (1). The centrality measures used for analysis in this research work are discussed below.

### Degree centrality

Regarded as a cornerstone metric, degree centrality is fundamentally a quantitative measure that enumerates the ties a node maintains within the network. This attribute serves as an indicator of a node’s relational intensity and is mathematically formalized in Eq. (1).

(1) \begin{eqnarray*}Cen{t}_{Deg}^{i}=degree \left( i \right) .\end{eqnarray*}

### Closeness

Closeness is the length-based measure which calculates the average shortest distance of the node. The closeness centrality of a node i is shown in Eq. (2).

(2) \begin{eqnarray*}Cen{t}_{Clo}^{i}= \frac{1}{\sum _{j}dist \left( j,i \right) } \end{eqnarray*}

where dist(j,i) is distance between the nodes i and j.

### Betweenness

Betweenness is the quantity-based measure which calculates the number of times the node acts a bridge along the shortest path between the nodes. The nodes which are most influential will have the highest betweenness value. The betweenness centrality of a node i is shown in Eq. (3).

(3) \begin{eqnarray*}Cen{t}_{Bet}^{i}=\sum _{i\not = j\not = k\in V} \frac{NS{P}_{ik} \left( V \right) }{NS{P}_{ik}} \end{eqnarray*}

where $NS{P}_{ik} \left( V \right) $ is the total number of shortest path from i to k.

*NSP*_*ik*_ is the number of paths through i.

### Eigen vector centrality

A sophisticated metric, Eigenvalue centrality serves as a gauge for a node’s propensity to wield influence within a network structure. Perhaps the most emblematic application of Eigenvalue centrality variations is Google’s PageRank algorithm, a seminal technique in search engine optimization. Mathematically, the Eigenvalue centrality of a node i is delineated in Eq. (4) as follows:

(4) \begin{eqnarray*}Cen{t}_{Eig}^{x} \left( i \right) = \frac{1}{\alpha } \sum _{j\in N \left( x \right) }{x}_{j}\end{eqnarray*}

where N(x) represents the ensemble of neighboring nodes connected to *x*, and *α* is a constant scaling factor.

## Model selection

Community detection is a sophisticated endeavor aimed at identifying nodes with elevated degrees of connectivity and assessing the extent of their influence within the network fabric. The crux of this exploration hinges on a model-based approach designed to delineate clusters or communities, anchored by their interrelational ties.

### Conventional model for two-mode networks

The two-mode network has the data in the *m x n* matrix with the occurrences of observation_ij_, where m individuals and n events. The observation_ij_ is the binary variable, where O_ij_ = 1, if tie exist and O_ij_ = 0, otherwise. Assumption of starting with X number of individuals in the community having the community extent ce = (ce_1_, ce_2_, …, ce _X_). The method will group the individuals having the common tie with the events. The conventional model is best suited only for the networks which are mutually exclusive and satisfies the following conditions as discussed by Aitkin, Vu & Francis (2017).

Condition 1: *ce*_*x*_ ≥ 0, for each x

Condition 2: ${\mathop{\sum }\nolimits }_{x=1}^{X}c{e}_{x}=1$.

### Multiple criteria model (MCM) framework

The multiple criteria model (MCM) serves as an intricate framework optimized for decision-making in scenarios fraught with multidimensional events and heterogenous observations, each characterized by a diverse array of attributes and parameters. Requiring domain-specific expertise, the MCM exhibits multidisciplinary versatility. Within the context of social network analysis, the MCM emerges as a robust tool, uniquely qualified to architect networks by navigating a plethora of observational choices. It adeptly identifies influential nodes, prioritizing them based on a pre-determined set of criteria. Notably, the scholarly landscape is replete with a variety of MCM paradigms, each tailored for specific decision-making applications.

ELECTRE (Saracoglu, 2015) and PROMETHEE P (Velasquez & Hester, 2013) serve as cornerstone methodologies in the realm of multi-criteria decision-making, operating on the principle of outranking. These paradigms are particularly apt for scenarios characterized by a limited set of criteria but an expansive array of alternatives. Conversely, the analytical hierarchy process (AHP) (Rios & Duarte, 2021) adopts a structured approach, employing pairwise comparisons to discern the most advantageous alternative. The Pugh method (Zhu et al., 2022), also known as the decision matrix method (DMM), takes a qualitative stance, benchmarking alternatives against a datum option. In the quantitative spectrum, the Statistical Design Institute (SDI) (Miranda, Antunes & Gama, 2022) computes scores for each design option. Technique for Order Preference by Similarity to an Ideal Solution (TOPSIS) (Ogonowski, 2022) emerges as an alternative to ELECTRE, specifically designed to pinpoint alternatives that closely approximate an ideal solution.

The quest to identify influential nodes within networks introduces a complex multi-criteria decision-making conundrum, rooted in the interplay between nodal influence weights and intricate topological attributes. This intricate dilemma serves as the intellectual impetus behind the development of our pioneering approach. Employing a fuzzy-based multiple criteria and multiple constraint level paradigm, this methodology innovatively harnesses grading techniques and community detection algorithms for the precise identification of influential nodes.

## Analytical Framework for Bipartite Insurgent Network Dissection

**Figure 3:**
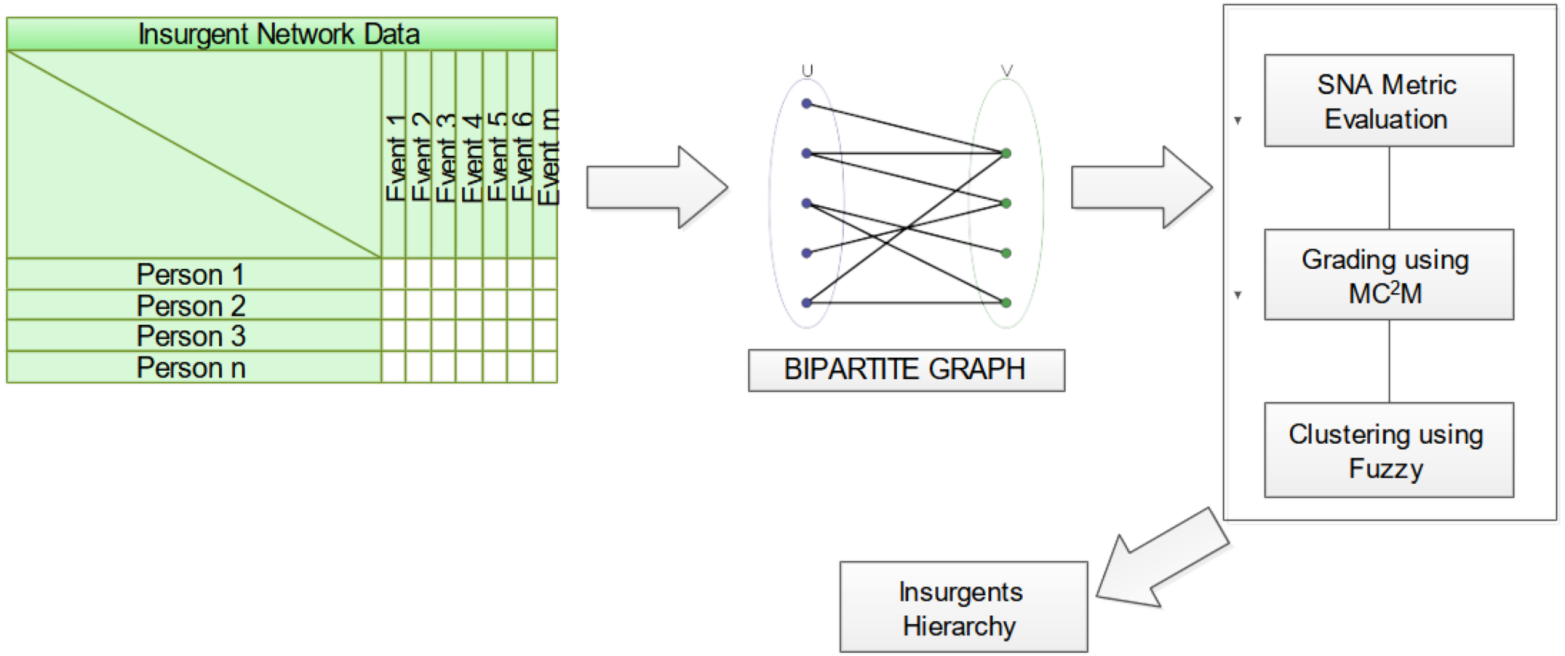
Schematic diagram of proposed approach for insurgent network analysis.

The methodology advanced herein focuses on the intricate analysis of two-mode insurgent networks, mathematically captured as a bipartite graph *G*_*b*_ = *{T,E,R}*, where *T* constitutes the ensemble of terrorists, *E* the array of events, and *R* the relational linkage intertwining *T* and *E*. Figure 3 elucidates the structural blueprint of this novel proposition.

The analytical ingress point is a data matrix, its rows personifying individuals purporting to be insurgents and its columns enumerating events in which these individuals partake. Matrix entries denote the individual’s engagement in a specific event. The inaugural phase entails the transformation of this data matrix into a bipartite graph. Subsequently, network attributes are calculated leveraging the Jaccard coefficient, a measure elucidating the neighborhood similarity between vertices, thereby enhancing object detection fidelity. Building on this foundation, the proposed multi-criteria and multi-constraint level approach is employed for node ranking through similarity score computations. Fuzzy-based clustering techniques are applied to demarcate communities, guided by the aforementioned metrics. Culminating this analytical odyssey, individual nodes are hierarchically positioned within these communities based on the gradings computed. Finally, the ascertained influential nodes, characterized by their community-specific gradings, offer invaluable insights into the behavioral patterns of the involved insurgents.

Finally, from the set of influential nodes with the grading of nodes in community helps to track the nature of insurgents involved in the activity.

## Comprehensive Execution of FMC^2^ Model for Analyzing Insurgent Networks

**Figure 4:**
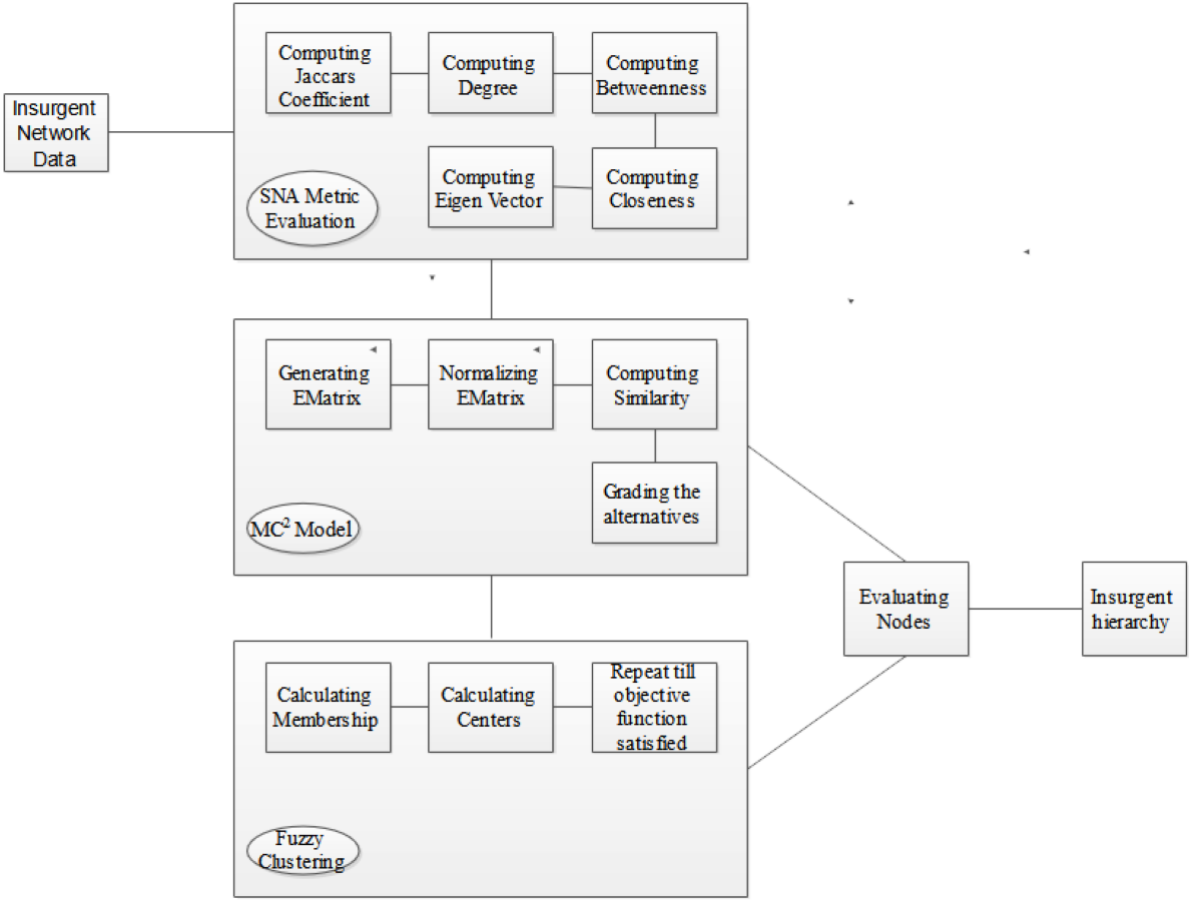
Implementation procedure of FMC2 model for insurgent network analysis.

This section meticulously delineates the procedural architecture underpinning the implementation of our avant-garde fuzzy multiple criteria multiple constraint model (FMC2) tailored for insurgent network scrutiny. Figure 4 provides a graphical exposition of the sequential steps implicated in the deployment of the FMC^2^ framework.

The fulcrum of the proposed model is to engender a hierarchical taxonomy of nodes, contextualized by their event-centric involvements, thereby facilitating the demarcation of insurgents into top, middle, and bottom tiers. The analytical journey commences with the construction of a bipartite graph derived from the input network dataset. Algorithm 1, encapsulating the FMC^2^ methodology, ingests this graph to compute the Jaccard coefficient; this is conditioned by the bipartite partitioning values, which are in turn modulated by event-specific involvements. Concomitantly, a compendium of network centrality measures—degree, closeness, betweenness, and eigenvector centrality—is calculated, each nuanced by the Jaccard coefficient. Following this, an evaluation matrix (EMatrix) is synthesized, encapsulating these computed metrics as criteria against a predefined set of alternatives. This matrix undergoes a weighted average normalization procedure, subsequent to which the extremal alternatives (best- and worst-case scenarios) are determined *via* shortest-path computations. Distances for each alternative are then quantified, serving as the basis for similarity score calculations that ultimately grade these alternatives.

At this juncture, the influential network nodes are discernibly identified *via* the computed metrics. The algorithm uniquely accommodates multiple criteria and constraints in a unified analytical framework. Finally, EMatrix is reevaluated, and fuzzy memberships µ_*i*_ and fuzzy centers are iteratively computed until the objective function converges, subject to *n* observations and *k* clusters. Additionally, *dsim(i,j)* signifies the dissimilarity measure between observations, and *e* represents the membership exponent

Algorithm 1: FMC^2^ Method

Input:

Bg- bipartite graph

Output:

Comm –Set of Clusters

Comm_infl –Community wise Influential nodes in hierarchical position

Method:

procedure FMC^2^Approach (Bg)

{

Bp =bipartite_partition_binary(Bg)

for each vertex in Bp{

set Bp_11_ = x, Bp_10_ = y, Bp_01_ = z

$JS \left[ i \right] = \frac{x}{x+y+z} $

if(JS[i]> 0.95)

 pJaccard[i] = 1

else

 pJaccard[j] = 0

diagonal of pJaccard = 0

$Cen{t}_{Deg}^{i}=degree \left( i \right) Cen{t}_{Clo}^{i}= \frac{1}{{\sum }_{j}dist \left( j,i \right) } Cen{t}_{Bet}^{i}={\sum }_{i\not = j\not = k\in V} \frac{NS{P}_{ik} \left( V \right) }{NS{P}_{ik}} Cen{t}_{Eig}^{x} \left( i \right) = \frac{1}{\alpha } {\sum }_{j\in N \left( x \right) }{x}_{j}$

}

Generate EMatrix_nxm_

Calculate EMatrix_Norm_nxm_ for each choice ${n}_{ij}= \frac{EMatri{x}_{ij}}{\sqrt{\sum EMatri{x}_{ij}^{2}}} $

$W={ \left( {w}_{ij} \right) }_{nxm}={ \left( {a}_{j}{n}_{ij} \right) }_{nxm}{S}_{w}=distance{S}_{b}=distanc{e}_{iw}= \frac{{S}_{w}}{{S}_{w}+{S}_{b}} $

Comm_infl = Grade (_*iw*_

Comm = ${F}_{min}={\mathop{\sum }\nolimits }_{c=1}^{k} \frac{{\mathop{\sum }\nolimits }_{i,j=1}^{n}{\mu }_{ic}^{e}{\mu }_{jc}^{e}dsim \left( i,j \right) }{2{\mathop{\sum }\nolimits }_{j=1}^{n}{\mu }_{jc}^{e}} $

}

## Simulation Result

In this section, simulation carried out using ‘R’ (Murdoch, 2017) for the proposed work is discussed and the results are reported.

### Dataset utilization: analyzing noordin top’s terrorist network

In the present study, we employ the Noordin Top Terrorist Network dataset (International Crisis Group, 2006), a publicly accessible corpus curated by the International Crisis Group. This dataset encapsulates data on 79 individuals implicated in extremist activities, with a specific focus on operations staged in Jakarta and Bali, Indonesia, in the year 2011. The dataset is comprehensive, encompassing a total of 568 distinct events, some of which wield significant influence on the affiliative dynamics within extremist organizations. The scope of affiliations scrutinized in our analysis spans a diverse range of institutions—educational establishments (schools and colleges), commercial enterprises, and religious organizations—as well as an array of relational dimensions such as classmate interactions, familial ties, friendships, co-religious affiliations, and other logistical support networks involved in training, terrorist activities, and strategic assemblies..

### SNA metric evaluation—quantifying node attributes in the network

Table 1 delineates a suite of computational metrics—namely, degree, betweenness, closeness, and eigenvector centrality—applied to the 79 nodes constituting the network under study. The “degree” serves as an indicator of a node’s connectivity within the network, quantifying the number of edges emanating from or converging to it. “Betweenness” furnishes insights into a node’s role as a connective bottleneck or gateway, bridging disparate clusters within the network. The “closeness” metric elucidates the extent to which a node is intimately connected to others in the network, essentially serving as a measure of its reachability. Lastly, “eigenvector centrality” offers a nuanced understanding of a node’s influence, taking into account not merely the quantity but the quality of its connections.

**Table 1:** Summary of centrality measures of the noordin data set.

**Person ID**	**Degree**	**Betweeness**	**Closeness**	**Eigen centrality**
1	222	272.8423	0.0029	0.8294
2	212	272.5252	0.0028	0.7642
3	220	272.7874	0.0028	0.8136
4	219	272.6826	0.0028	0.8093
5	243	275.1958	0.0030	0.9489
6	216	272.6378	0.0028	0.7870
7	210	272.5108	0.0028	0.7517
8	214	272.5477	0.0028	0.7767
9	214	272.5717	0.0028	0.7803
10	209	272.5063	0.0028	0.7453
11	214	272.5676	0.0028	0.7754
12	209	272.5063	0.0028	0.7460
13	220	272.7990	0.0028	0.8128
14	214	272.5717	0.0028	0.7803
15	234	273.9582	0.0030	0.8983
16	217	272.6415	0.0028	0.7992
17	209	272.5063	0.0028	0.7449
18	217	272.6627	0.0028	0.7970
19	214	272.5647	0.0028	0.7792
20	215	272.5992	0.0028	0.7864
21	214	272.5635	0.0028	0.7800
22	216	272.6213	0.0028	0.7889
23	244	275.4381	0.0030	0.9532
24	232	273.7902	0.0029	0.8823
25	209	272.5063	0.0028	0.7460
26	216	272.6120	0.0028	0.7911
27	211	272.5188	0.0028	0.7603
28	212	272.5249	0.0028	0.7645
29	213	272.5467	0.0028	0.7727
30	218	272.7110	0.0028	0.8044
31	226	273.1580	0.0029	0.8485
32	213	272.5510	0.0028	0.7726
33	210	272.5108	0.0028	0.7517
34	212	272.5339	0.0028	0.7648
35	214	272.5728	0.0028	0.7796
36	220	272.8028	0.0028	0.8170
37	216	272.5846	0.0028	0.7910
38	209	272.5063	0.0028	0.7452
39	224	272.9949	0.0029	0.8361
40	209	272.5063	0.0028	0.7446
41	216	272.6311	0.0028	0.7927
42	217	272.6648	0.0028	0.7983
43	211	272.5153	0.0028	0.7591
44	214	272.5754	0.0028	0.7763
45	230	273.5313	0.0029	0.8718
46	237	274.4094	0.0030	0.9111
47	212	272.5317	0.0028	0.7671
48	210	272.5105	0.0028	0.7531
49	214	272.5717	0.0028	0.7803
50	225	273.0542	0.0029	0.8451
51	214	272.5533	0.0028	0.7769
52	219	272.7466	0.0028	0.8074
53	212	272.5198	0.0028	0.7655
54	211	272.5193	0.0028	0.7598
55	217	272.6642	0.0028	0.7987
56	228	273.3755	0.0029	0.8623
57	223	272.9501	0.0029	0.8346
58	212	272.5322	0.0028	0.7666
59	252	276.8691	0.0031	1.0000
60	216	272.5995	0.0028	0.7902
61	211	272.5198	0.0028	0.7586
62	212	272.5317	0.0028	0.7671
63	224	273.0142	0.0029	0.8393
64	227	273.2198	0.0029	0.8546
65	215	272.5992	0.0028	0.7864
66	213	272.5506	0.0028	0.7729
67	210	272.5063	0.0028	0.7527
68	215	272.6024	0.0028	0.7832
69	209	272.5063	0.0028	0.7460
70	224	273.0291	0.0029	0.8370
71	213	272.5520	0.0028	0.7707
72	208	272.5063	0.0027	0.7384
73	214	272.5753	0.0028	0.7762
74	213	272.5411	0.0028	0.7734
75	209	272.5063	0.0028	0.7451
76	212	272.5322	0.0028	0.7666
77	211	272.5154	0.0028	0.7580
78	210	272.5109	0.0028	0.7516
79	210	272.5110	0.0028	0.7513

### FMC^2^ based perception grading method

The similarity score computed by applying the proposed FMC^2^ method and the grade based on the computed score are depicted in the Table 2.

**Table 2:** Summary of similarity score and grade evaluated for Noordin network.

**ID**	**Score**	**Grade**	**ID**	**Score**	**Grade**	**ID**	**Score**	**Grade**
1	0.3343	16	28	0.0956	58	55	0.2194	24
2	0.0948	59	29	0.1238	49	56	0.4631	8
3	0.2793	18	30	0.2412	22	57	0.3549	15
4	0.2609	20	31	0.4129	10	58	0.1010	54.5
5	0.7984	3	32	0.1235	50	59	1.0000	1
6	0.1824	32	33	0.0483	67.5	60	0.1904	30
7	0.0483	67.5	34	0.0963	57	61	0.0732	63
8	0.1412	43	35	0.1486	40	62	0.1023	52.5
9	0.1505	37	36	0.2880	17	63	0.3746	12
10	0.0248	74	37	0.1924	29	64	0.4361	9
11	0.1380	46	38	0.0245	75	65	0.1734	33.5
12	0.0265	72	39	0.3666	14	66	0.1245	48
13	0.2775	19	40	0.0229	78	67	0.0510	66
14	0.1505	37	41	0.1968	27	68	0.1652	35
15	0.6001	5	42	0.2182	25	69	0.0265	72
16	0.2206	23	43	0.0745	62	70	0.3689	13
17	0.0238	77	44	0.1404	44	71	0.1188	51
18	0.2150	26	45	0.5024	7	72	0.0000	79
19	0.1478	41	46	0.6560	4	73	0.1400	45
20	0.1734	33.5	47	0.1023	52.5	74	0.1256	47
21	0.1498	39	48	0.0521	65	75	0.0242	76
22	0.1869	31	49	0.1505	37	76	0.1010	54.5
23	0.8176	2	50	0.3967	11	77	0.0716	64
24	0.5446	6	51	0.1418	42	78	0.0482	69
25	0.0265	72	52	0.2563	21	79	0.0473	70
26	0.1926	28	53	0.0981	56			
27	0.0776	60	54	0.0763	61			

**Figure 5:**
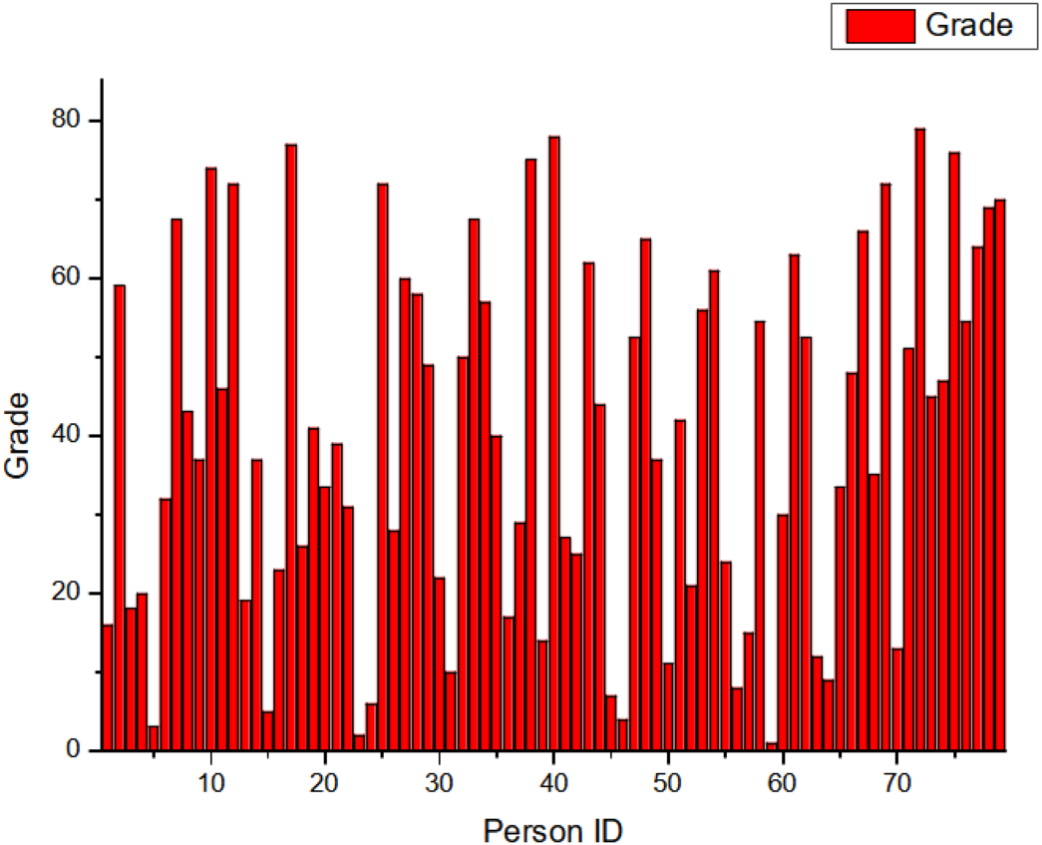
Grade distribution.

The scores, delineated in Fig. 5, manifest as fuzzy-based values in the interval [0,1][0,1], serving to classify the nodes on a continuum from least to most influential. The *x*-axis enumerates the Node IDs, while the *y*-axis represents the corresponding grade values. Notably, Node 59 emerges as an apex entity, registering a grade value of 1. Contrary to initial impressions, it should be emphasized that within the context of this network, a lower grade value paradoxically indicates heightened influence as opposed to a higher grade value.

### Fuzzy clustering

Fuzzy clustering, leveraging the output from the FMC^2^ model, initiates with a bifurcated cluster framework and iteratively expands to three clusters. Table 3 encapsulates the nuanced metrics: ***Dunn_Coeff***, representing the partition coefficient of the clustering, is normalized and designated as ***Normalized***. ***Obj_Func*** signifies the nadir of the objective function achieved through relative convergence tolerance, while the iterative count and average cluster width are also detailed.

**Figure 6:**
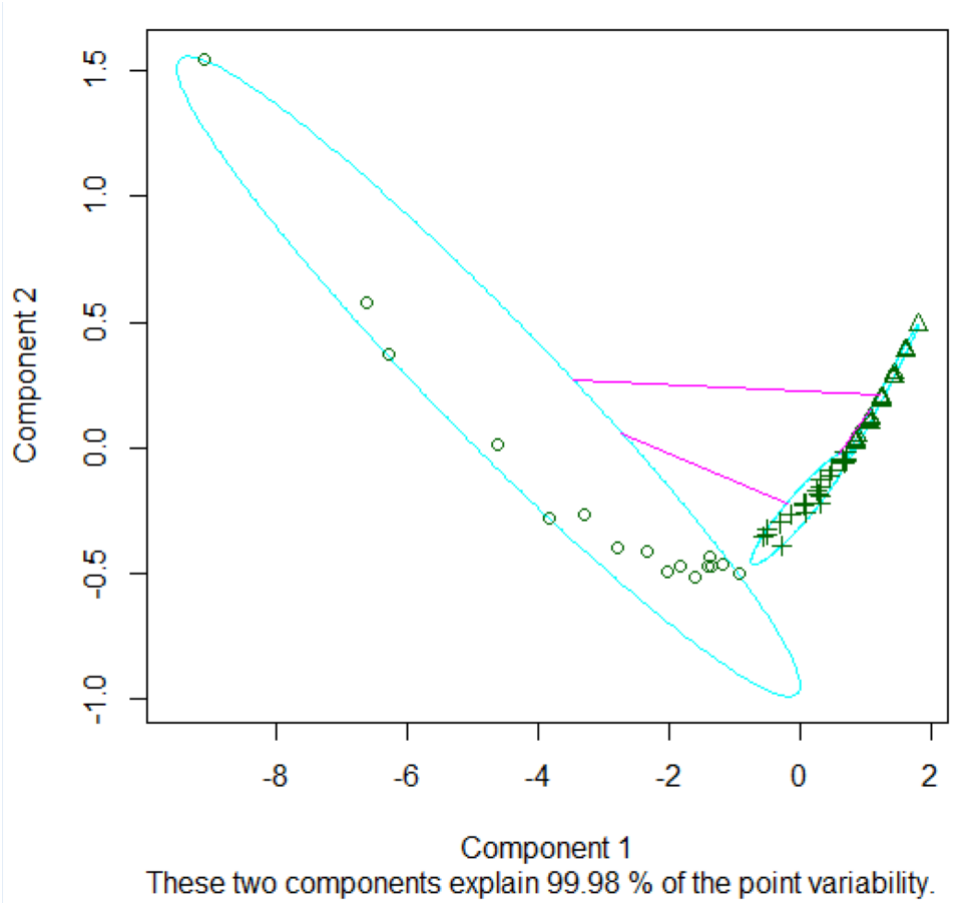
Clusters of *k* = 3.

Figure 6 delineates three distinct clusters, boasting cluster widths of 0.2555, 0.62588, and 0.53196, respectively. The constituent entities in these clusters are 16 for Cluster 1, 33 for Cluster 2, and 30 for Cluster 3.

 The ramifications of this clustering paradigm manifest as overlapping zones within the clusters, introducing what we term as ‘confusing actors’ in network analytics. These actors present a deceptive semblance of influence owing to the specific nature of their network ties, thereby complicating the analytical landscape.

**Figure 7:**
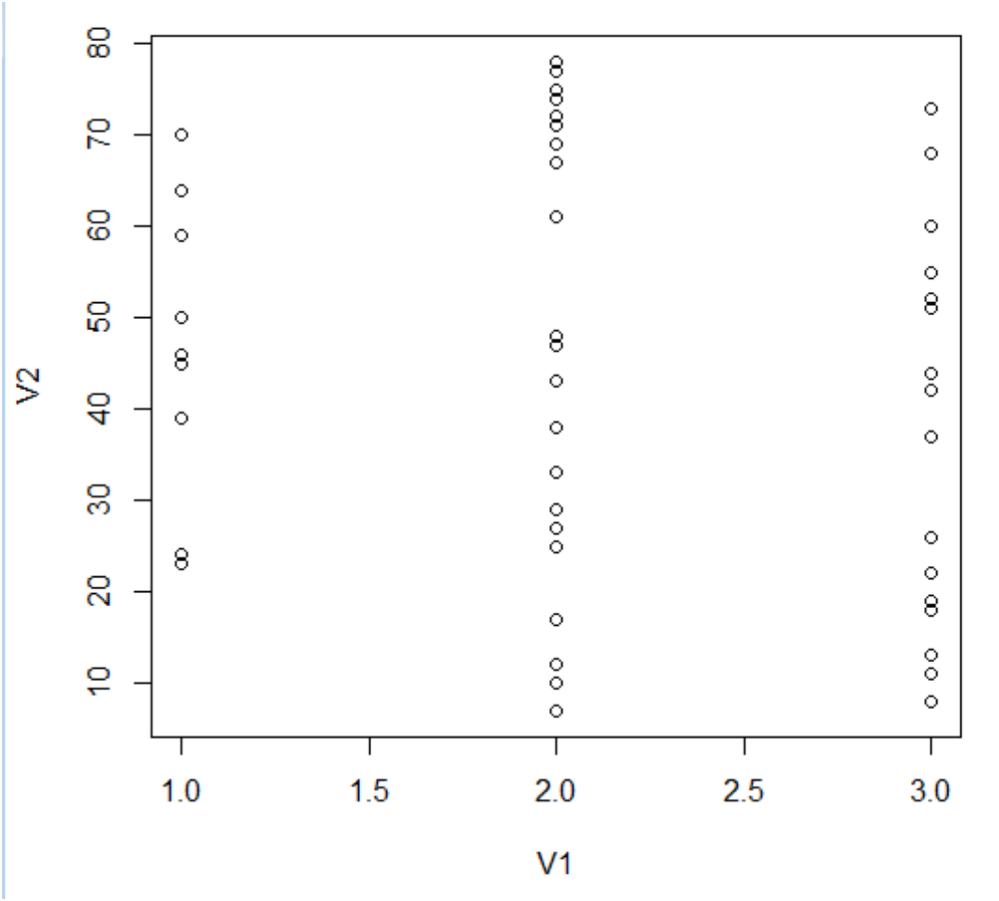
Structuring of cluster.

Figure 7 offers a visual representation of node clustering, wherein nodes are spatially organized based on their computed grades, set against their respective clusters.

**Table 3:** Summary of fuzzy clustering.

**Number of cluster**	**Dunn_Coeff**	**Normalized**	**Obj Func**	**Iterations**	**Average width**
2	0.99185	0.98371	184.9	30	0.704906
3	0.98789	0.98184	133.9	22	0.5152

Table 4 promulgates the hierarchical ranking of nodes within these clusters, serving as a testament to the efficacy of the proposed method.

**Table 4:** Summary of grade for nodes in clusters.

**Cluster 1**		**Cluster 2**		**Cluster 3**	
**Node**	**Grade**	**Node**	**Grade**	**Node**	**Grade**
59	1	13	10	74	26
23	2	52	11	29	27
46	3	55	12	71	28
24	4	42	13	47	29
45	5	18	14	27	30
64	6	26	15	43	31
50	7	37	16	61	32
70	8	60	17	77	33
39	9	22	18	48	34
		68	19	67	35
		19	20	7	36
		51	21	33	37
		8	22	78	38
		44	23	12	39
		73	24	25	40
		11	25	69	41
				10	42
				38	43
				75	44
				17	45
				72	46

Table 4 illuminates a structured hierarchy of insurgent actors, providing an intricate mapping of their relative import within terrorist activities in Indonesia. Arising from the application of our avant-garde methodology, we delineate the terrorist network into three concentric tiers:

(a) The central nexus comprises the preeminent figures who wield unparalleled authority and orchestrate the attacks with dictatorial command.

(b) The intermediate echelon consists of influential personas functioning as conduits between the central authority and the periphery. They are tethered to the central figures *via* operational and relational channels.

(c) The peripheral cadre, while less influential, serve as the logistical backbone to the operation and lack direct affiliations with the central authority.

**Figure 8:**
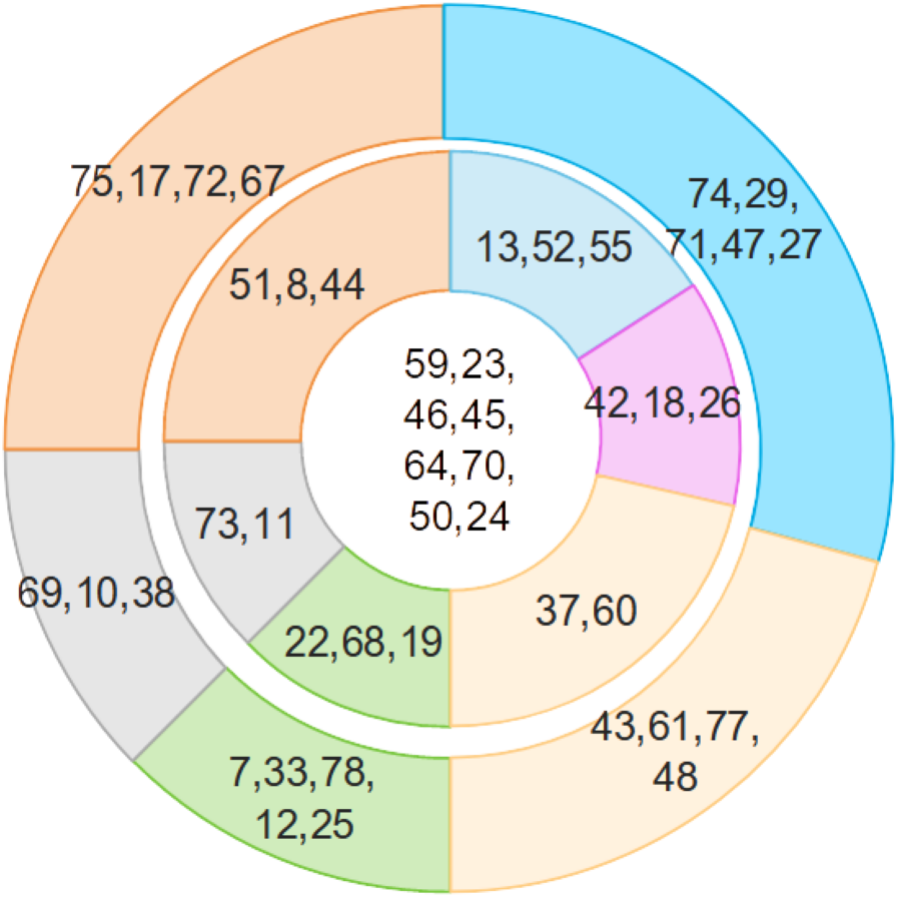
Hierarchies of insurgents.

Figure 8 graphically encapsulates this hierarchical stratification, offering a visually compelling elucidation of the intricate layers of insurgent influence.

### Performance analysis: empirical evaluation on the Noordin top terrorist dataset

Table 5 furnishes a rigorous quantitative assessment of our proposed algorithm’s efficacy in identifying influential nodes and effecting data clustering within the Noordin Top Terrorist Network dataset. The performance indicators encapsulated therein include sensitivity, specificity, positive predictive value (PPV), and negative predictive value (NPV).

**Table 5:** Performance of the categorization of persons in the Noordin network.

		**Data set**		**Sensitivity**	**Specificity**	**Positive predicted value**	**Negative predicted value**
		**Terrorist**	**Not terrorist**				
**Implementation result**	**Terrorist**	46	4	0.9787	0.875	0.92	0.9655
	**Not terrorist**	1	28				

*Sensitivity*: This metric quantifies the algorithm’s proficiency in accurately pinpointing nodes of genuine influence within the network.

*Specificity*: Complementary to sensitivity, this measure assesses the algorithm’s capability to correctly identify nodes that are not influential, thereby mitigating the risk of false positives. Positive predictive value (PPV) & negative predictive value (NPV): These values offer an enhanced understanding of the algorithm’s predictive precision, calibrated against the overall prevalence of influential nodes within the network.

## Conclusion and Future Work

In this article, we have introduced a groundbreaking methodology leveraging a fusion of fuzzy-based multi-criteria and multi-constraint mechanisms for grading nodes, thereby enabling the identification of influential actors in insurgent networks. Employing a suite of social network analysis metrics—namely, degree, betweenness, closeness, and eigenvector centrality—our approach harmonizes these conventional measures with fuzzy membership and modularity metrics to formulate a sophisticated community detection model. This facilitates the nuanced clustering of nodes within the complex structure of insurgent networks.

Our fuzzy multiple criteria multiple constraint level approach (FMC2) serves as the computational backbone for this undertaking. By computing similarity measures through a distance-based MC2 method, the FMC2 framework yields graded values that not only identify but also hierarchically classify influential nodes within the network. Furthermore, our methodology incorporates fuzzy boundaries in centrality-based measures, enhancing the precision of the resultant clusters.

This innovative approach is explicitly tailored for two-mode insurgent networks, accommodating multiple criteria and constraints concurrently for a more comprehensive analysis. However, its applicability is not confined to this specific type of network; it can be readily extended to other two-mode network structures. Looking ahead, future variations of this work could integrate machine learning techniques into our existing framework, offering yet another dimension of analytical depth.

## Supplemental Information

10.7717/peerj-cs.1644/supp-1 Supplemental Information 1 Implementation Code of the proposed approach

10.7717/peerj-cs.1644/supp-2 Supplemental Information 2 Main dataset for bipartite graph

10.7717/peerj-cs.1644/supp-3 Supplemental Information 3 Evaluation matrix for the algorithm generated from WorkingData.csv

10.7717/peerj-cs.1644/supp-4 Supplemental Information 4 Final cluster formation for WorkingData.csv by means of evaluation matrix using fuzzy approach

10.7717/peerj-cs.1644/supp-5 Supplemental Information 5 Code snippet

## Additional Information and Declarations

### Data Availability

The following information was supplied regarding data availability: The code and raw data are available in the Supplemental Files.
