# Supplementary material for: FMC2 model based perception grading for dark insurgent network analysis
Source: PeerJ Comput Sci. 2023 Dec 5;9:e1644. doi: 10.7717/peerj-cs.1644 (PMC10773563; doi:10.7717/peerj-cs.1644)
Supplement: Supplemental Information 1 [file peerj-cs-09-1644-s001.pdf]

```
library(igraph)

A <- read.csv(file="WorkingData.csv",header=F,sep=",")

M<-as.matrix(A)

nr=nrow(M)

nc=ncol(M)

g1 <- graph_from_incidence_matrix(M,directed=FALSE,mode="out",weighted=TRUE)

#Constructing Bipartite Graph

bipartite.mapping(g1)

V(g1)$type<-bipartite_mapping(g1)$type

plot(g1,layout=layout.bipartite)

types <- V(g1)$type

#Calculating Degree, betweenness, closeness, eigen centraility measures

deg<- degree(g1)

bet<-betweenness(g1)

clos<-closeness(g1)

eig<-eigen_centrality(g1)$vector

cent_df<-data.frame(types, deg, bet, clos, eig)

cent_df[order(cent_df$type,decreasing=TRUE),]

library(ade4)

bipartite_matrix<-as_incidence_matrix(g1)

#Calculating Jaccard cofficient and measures such as Degree, betweenness, closeness, eigen
#centraility measures

person_jacc<-dist.binary(bipartite_matrix,method=1,upper=TRUE,diag=FALSE)
```

```
org_jacc<-dist.binary(t(bipartite_matrix),method=1,upper=TRUE,diag=FALSE)

person_jacc<-as.matrix(person_jacc)

jacc_person<-ifelse(person_jacc>0.95,1,0)

diag(jacc_person)<-0

jacc_person

jacc_person<-graph_from_adjacency_matrix(jacc_person,mode="undirected")

plot(jacc_person)

person_deg<-degree(jacc_person)

person_bet<-betweenness(jacc_person)

person_clos<-closeness(jacc_person)

person_eig<-eigen_centrality(jacc_person)$vector

person_cent_df<-data.frame(person_deg, person_bet, person_clos, person_eig)

library(topsis)

#Evaluation matrix for clustering and grading

A <- read.csv(file="topsi.csv",header=F,sep=",")

m<-as.matrix(A)

w<-c(1,1,1,1)

i<-c("+","+","+","+")

top<-topsis(m,w,i)

top_df<-data.frame(top$alt.row,top$score,top$rank)

cwt <- cluster_walktrap(g1,weights=E(g1)$weight,steps=6)

library(cluster)

IMat<-m
```

```
cfuz<-fanny(IMat,2,memb.exp=1.1)

names(cfuz)

plot(cfuz)

fmem<-cfuz$membership

fcoeff<-cfuz$coeff

fmembexp<-cfuz$memb.exp

fclust<-cfuz$clustering

fkcrisp<-cfuz$k.crisp

fobj<-cfuz$objective

fconv<-cfuz$convergence

fdiss<-cfuz$diss

fcall<-cfuz$call

fsilinfo<-cfuz$silinfo

fdata<-cfuz$data

AC <- read.csv(file="Cluster.csv",header=F,sep=",")

MAC<-as.matrix(AC)

g<-graph(MAC)

plot(MAC)

cwt(g)

cwt <- cluster_walktrap(g,weights=E(g)$weight,steps=10)
```
